# Editorialets

**Published:** 1906-02

**Authors:** 


### Editorialets.

THE GULLIBLE PUBLIC.—"Rattle Snake Pete," Rochester, N. Y., is the latest to offer a sure cure for consumption. He gives the pulsating heart of a rattler. He is exploited in the Washington *Post* January 7th. A woman lecturer at Seattle, Wash., induced most of her audience to swallow a teaspoonful of black sand as a cure for indigestion. They all had to have a doctor to relieve the cramps.

DEATH OF DR. BRIGGS.—Dr. W. B. Briggs, of Easterly, Texas, the well known and popular Secretary of the Brazos Valley Medical Association for so many years, was found dead in his room about the first of January. There is no way of finding out just when or how he died, as he lived alone. He had stated that he was going away to visit some relatives, and when his house was found locked, nothing was thought of it. He had been dead many days when his body was found. I have not been able to get any biographical data of him except that he was a Tennesseean, an M. D. from the medical department, University of Nashville, class of 1862, and was a Confederate surgeon. Dear old Briggs, genial and companionable and as full of fun as a boy. We will miss him. He left no family. When he sent out the notice for the meeting of the Brazos Valley Medical Society, May, 1897, he wrote at the top of it:

"Like as a plank of driftwood tossed on the watery main,
One plank encounters, meets, touches, parts again;
'Tis thus with friends forever, on life's uncertain sea,
They meet, and greet, and sever, drifting eternally."

He has drifted into the Great Beyond and we will meet him no more.

---

I TOLD YOU SO.—I said that one function of the journals of the State Associations was to be feeders of the Octopus; to whoop up subscriptions for it; and whip in members for the A. M. A. Here is proof of it. This appeared in the January issue of the Texas Tentacle, and was doubtless dictated or sent from Chicago. It didn't originate with Dr. Chase—for in the second paragraph, produced below, he kicks,—but he just had to:

*The Journal of the A. M. A.*—Every member of the State Medical Association of Texas should be a subscriber to the *Journal of the A. M. A.* It is the greatest medical journal in the world. Its bound volumes furnish the most complete medical references obtainable. Every member of the State Medical Association should likewise be a member of the American Medical Association. The *Journal* costs $5 a year, and all subscribers who are members of a State association can become a member of the national organization on request, and without extra expense. The *Journal* represents the American medical profession, and as such is the champion of national medical reforms, and worthy of our support.

Texas now stands twelfth among the States in the number of subscribers to this journal. There are about 1100 Texas members of the A. M. A., which is approximately twice as many as from any other southern State. Before the end of 1906 we hope to see this number increased to 2000, that is, until two-thirds of the members of the State Medical Association of Texas are enrolled. Eleven hundred gives a fair estimate of the most progressive of the 5000 physicians in the Texas State profession.—*Texas State Journal of Medicine,* January, '06.

*Octopus vs. Star Fish.*—One of our Councilors writes calling our attention to advertisements in the *Journal of the A. M. A.* of Campho-Phenique, Bovinine, Pepto-Mangan and other semi-secret proprietary remedies, doubtless possessing therapeutic virtue, but unfortunately now classed among the nostrums which the Board of Trustees do not admit among the advertising pages of this *Journal.* He says: "I fail to see wherein the 'Octopus' may wedge in these advertisements and the 'Starfish' be deprived of this sustenance."

It is unfortunate that the State journals can not have an illustrious example set them by the great *Journal of the A. M. A.* in matters of clean advertising. We, however, have the assurance from the Board of Trustees that as soon as contracts expire, all objectionable advertising will disappear from the pages of our national publication. — *Texas State Journal of Medicine,* January, '06.

The Octopus runs with the hare for revenue, and holds with the hounds for bumcombe.

---

WITH the new year *The Alkaloidal Clinic* rises from its ashes as *The American Journal of Clinical Medicine.* The first number is a stunner. Editorial cabinet: Abbott, Waugh, Robinson (W. J.), Lanphear, the inimitable, Burdick, and Epstein. Drop a card to Dr. Abbott and tell him that I requested you to do so,—to send you a copy of the January number free. He'll *do* it.

---

AS TO ETHICS: CHICAGO DOCTOR WENT.—Chicago, Jan. 11.—Dr. Frank Billings, summoned to the sickbed of Marshal Field, left for New York this afternoon at 2:30 o'clock on the Twentieth Century Limited over the Lake Shore. He will arrive at the Holland House tomorrow morning.

CHICAGO DOCTOR DIDN'T GO.—Chicago, Jan. 12.—Dr. Frank Billings, who is Marshall Field's personal physician, denied last night that he had been summoned to New York. "I have received no intimation that my presence was desired by Mr. Field," said Dr. Billings last night. "The subject has been broached to me, however, by a number of his relatives and friends and it is possible that I may leave for New York city tomorrow. I am waiting to hear from Mr. Field before I make any preparations to leave."—*N. Y. Herald.*

And this is the man who couldn't stomach any kind of ready-to-dispense pharmacals: it is "unethical."

---

"THE BLOODLESS PHLEBOTOMIST" is the name of a handsome pamphlet issued by the Denver Chemical Co., proprietors of Antiphlogistine. Antiphlogistine is recognized by and incorporated into the U. S. P. as *Emp. Kaolin Com.* The original papers in the January issue are by the most distinguished authorities in the world; yet ye Octopus says that the Denver fellows must obey its orders or no go. They are as follows: "Appendicitis as an Infective Inflammation," by Prof. Robert T. Morris, A. M., M. D., of

New York; "The Early Diagnosis of Pulmonary Tuberculosis," by H. Edwin Lewis, M. D., of New York; "Phagedenic Ulcer," by J. Bonnefin, M. R. C. S., of Leytonstone, Eng.

---

Dr. J. A. Coffman, of Poetry, Texas, a graduate of the Kentucky School of Medicine, class of 1890, fell from a trestle at Terrell, Texas, January 19th, ult., and was killed.

---

Quarantine officers from Texas, Mississippi, Louisiana, and Alabama sailed from New Orleans January 20th (ult.) for the Central and South American ports whence the tropical fruits are shipped, for the purpose of looking into the sanitary condition of said ports and the condition under which fruit is loaded and shipped. I understand that this inspection was invited by the Fruit Shipping Company of New Orleans,—who will pay all expenses,—to refute the charge that the yellow fever was introduced into that city by the fruit ships. Dr. Jamie W. McLaughlin, Jr., of Austin, represents the State of Texas in the Commission.

---

"Sanitation at Panama and Colon." Poultney Bigelow, a newspaper correspondent, has raised a hornet's nest by exposing the unsanitary conditions at Colon. Mr. Roosevelt and Mr. Tafft say it "ain't so," but Congress has appointed a commission to go and see whether or not Mr. Bigelow is a "sensation monger," as Mr. Tafft says he is. Look out for squalls. "A chiel's among ye, taking notes and in faith, he'll print 'em."

---

The Moat and the Beam.—Billings, in his screed against all and singular proprietary pharmacals, spells "emmenagogue" with one "m," and calls all ready-to-give medicines "secret nostrums." That is about like saying "a bovine bull" or a "canine dog" or a "feline cat" or a "pyrotechnical display of fireworks" or a "subcutaneous incision under the skin," and is equal to saying *secret* "secret remedies" for a "nostrum" is a "secret remedy." Simmons says that "superstitious" is derived from the Latin *superstet*, and means "a survivor." Webster says it is derived from *superstare* ("super" and "stare"), and means to "stand over." I only mention it to show what a clam Webster is, compared to the Octopus fellow.

